# The U-Shaped Association of Non-High-Density Lipoprotein Cholesterol Levels With All-Cause and Cardiovascular Mortality Among Patients With Hypertension

**DOI:** 10.3389/fcvm.2021.707701

**Published:** 2021-07-14

**Authors:** Qi Cheng, Xiao-cong Liu, Chao-lei Chen, Yu-qing Huang, Ying-qing Feng, Ji-yan Chen

**Affiliations:** ^1^School of Medicine, South China University of Technology, Guangzhou, China; ^2^Department of Cardiology, Guangdong Cardiovascular Institute, Guangdong Provincial People's Hospital, Guangdong Academy of Medical Sciences, Guangzhou, China

**Keywords:** non-high-density lipoprotein cholesterol, hypertension, all-cause mortality, cardiovascular mortality, NHANES

## Abstract

**Background:** Non-high-density lipoprotein cholesterol (non-HDL-C) is a valuable indicator in routine blood lipid tests, but the associations of non-HDL-C with mortality in hypertensive population still remain uncertain.

**Methods:** In the National Health and Nutrition Examination Surveys from 1999 to 2014, participants having hypertension were included and grouped by non-HDL-C levels (<130, 130–159, 160–189, 190–219, and ≥220 mg/dl). Multivariate Cox regression was conducted for calculation of hazard ratios (HR) and 95% confidence interval (CI). To reveal the relationship between non-HDL-C and mortality, Kaplan–Meier survival curves, restricted cubic spline, linear regression, and subgroup analysis were also applied.

**Results:** A total of 12,169 participants (47.52% males, mean age 57.27 ± 15.79 years) were included. During average follow-up of 92.5 months, 1,946 (15.99%) all-cause deaths and 422 (3.47%) cardiovascular deaths occurred. After adjusting for confounders, the association of non-HDL-C with mortality was detected as U-shaped. Threshold values were observed at 158 mg/dl for all-cause mortality and 190 mg/dl as to cardiovascular mortality. Below the threshold, every 10 mg/dl increment in non-HDL-C attributed to relatively low all-cause mortality significantly (HR = 0.94, 95% CI: 0.92–0.96). Above the threshold, non-HDL-C has significant positive associations with both all-cause (HR = 1.03, 95% CI: 1.01–1.05) and cardiovascular mortality (HR = 1.09, 95% CI: 1.05–1.14). For subgroups analysis, similar results were found among participants age <65 years old, non-white population, those were not taking lipid-lowering drugs, and subjects with body mass index (BMI) ≥25 kg/m^2^.

**Conclusion:** The U-shaped association was detected between non-HDL-C and mortality among hypertensive population.

## Introduction

As reported by current epidemiological data, hypertension is a common chronic disease in humans, involving a population of more than 1.13 billion worldwide, and the global prevalence of hypertension is still growing (1). Generally, hypertension is not accompanied by obvious relevant symptoms, and patients can have hypertension without knowing it. Hypertension is like a silent killer, which causes various cardiovascular and cerebrovascular complications (1). Dyslipidemia is not only a risk factor for hypertension but also closely related to the prognosis of hypertension (2, 3). Research have found that interventions for blood pressure and cholesterol in hypertensive population could bring long-term benefits (4). Therefore, it is of importance to evaluate and monitor the blood lipid status of hypertensive patients for comprehensive prevention and control of hypertension (1, 5). Non-high-density lipoprotein cholesterol (non-HDL-C) is a parameter containing all kinds of lipoprotein cholesterol except high-density lipoprotein cholesterol (HDL-C) (6). The total cholesterol (TC) and HDL-C concentrations can be assessed by standardized detection methods; TC subtracting HDL-C can calculate non-HDL-C levels accurately and easily. Some studies claimed that the measure of apolipoprotein B (apoB) is more accurate for risk assessment (7). However, compared with apoB, non-HDL-C concentrations are easier to obtain by its simple calculation, requiring no additional costs. Besides, triglyceride (TG), the lipid factor that may be affected by diet, is not included in the calculation formula. Thus, the appliance of non-HDL-C will not increase experimental cost and medical expenses of patients, and it can even be tested in non-fasting state, which brings convenience to both medical staff and patients (8). Proven by a large number of researches, non-HDL-C was regarded as a reliable indicator for cardiovascular risk stratification (9, 10). Due to the multiple merits of non-HDL-C application mentioned above, the current guidelines have proposed non-HDL-C as an alternate parameter to low-density lipoprotein cholesterol (LDL-C) for monitoring lipid status (11–13).

Previously, the studies exploring associations of non-HDL-C and mortality had different outcomes. For example, a positive association was found in general population (14–16) or patients with cardiovascular disease (CVD) (17–19). However, a study in Mediterranean population with diabetes (20) and a study in elderly European males found no associations (21). Besides, some studies in patients with renal dysfunction showed that elevated non-HDL-C could predict higher cardiovascular mortality risk independently (22, 23), while another study found an inverse association (24). Similar inverse associations were also found in elderly (25, 26). Recently, a U-shaped relationship was also discovered (27). In hypertensive population, rising non-HDL-C could develop arterial stiffness (28, 29) and even accelerate the process of hypertension (30). Also, non-HDL-C levels were observed to be significantly higher in hypertensive patients compared with healthy population, indicating that non-HDL-C was a risk indicator worth studying in this concerned population (31). All in all, the pattern of how non-HDL-C associates with mortality is still controversial, and there are few studies investigating this association among hypertensive population. Thus, we intend to study the association of non-HDL-C with mortality among patients with hypertension by analyzing data from the large population representative surveys conducted in the United States.

## Methods

### Study Design and Population

**Figure 1:**
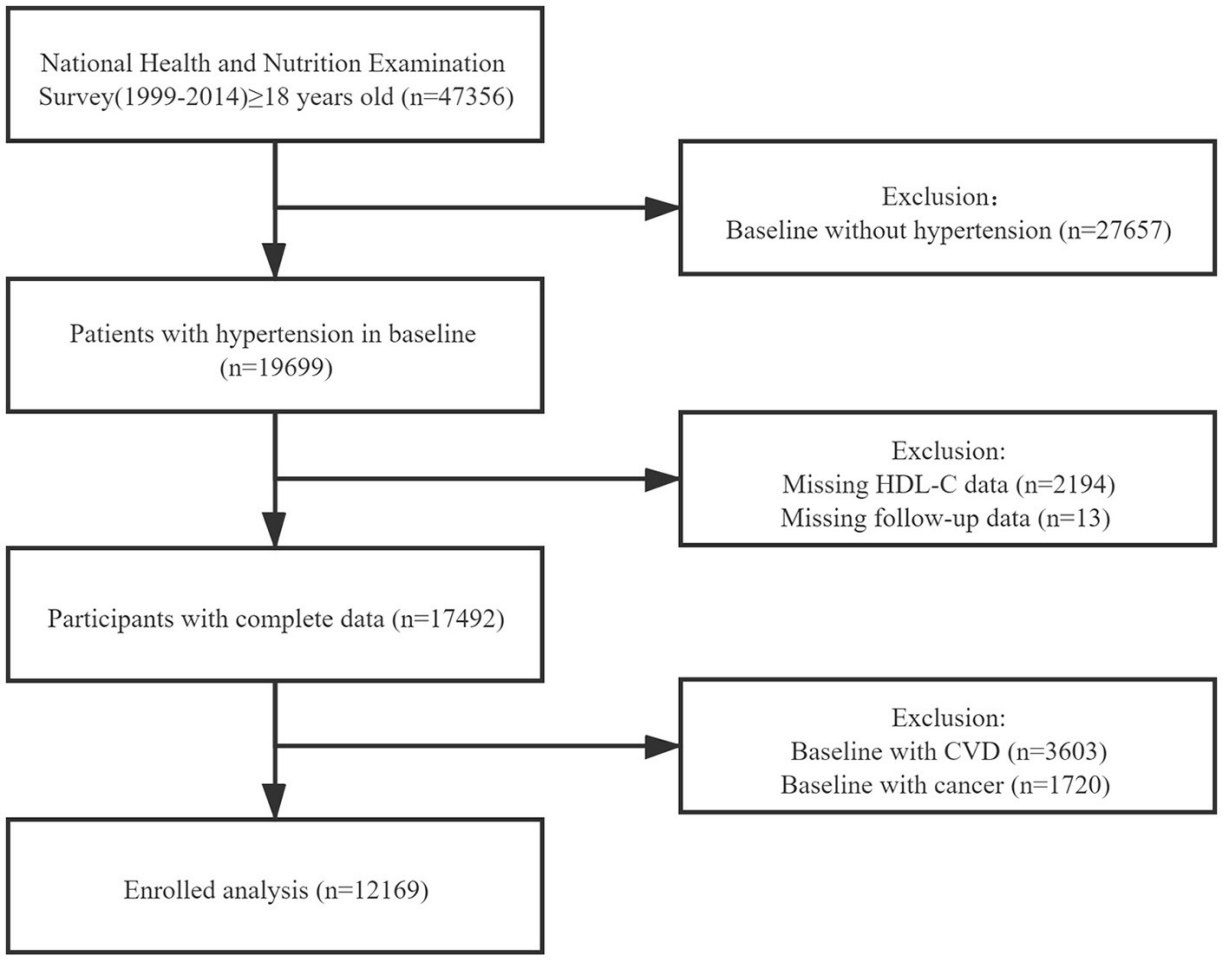
The research flow chart.

We extracted the data of study population from National Health and Nutrition Examination Surveys (NHANES) 1999-2014. NHANES, conducted by the National Center for Health Statistics, is a series of nationwide surveys to evaluate the health status of the US citizens. The Centers for Disease Control and Prevention has ratified the study protocols, and all participants have provided informed consents. The total number of the participants in the survey is 47,356. After excluding the participants in baseline without hypertension (*n* = 27,657), missing HDL-C data (*n* = 2,194) or follow-up data (*n* = 13), and those in baseline with CVD or cancer, a total number of 12,169 individuals were enrolled in our formal analysis (Figure 1).

### Exposure

The variable being exposed was non-HDL-C concentration. The collection of blood samples and lipid concentration measurement were based on standardized procedures. Hitachi 704 Analyzer (Boehringer Mannheim Diagnostics, Indianapolis, IN, USA) was applied to examine all the blood samples (32). HDL-C was obtained by direct immunoassay or a heparin-manganese precipitation method, while TG and TC were measured enzymatically (33). LDL-C was computed by the Friedewald formula when TG ≤ 400 mg/dl (34). Non-HDL-C was computed by TC subtracting HDL-C (13).

### Covariates

The covariates were assessed at baseline by using standardized questionnaires and physical examinations. These covariates included age, gender, race (non-white vs. white), smoking status (yes or no), diabetes (yes or no), body mass index (BMI), systolic blood pressure (SBP), diastolic blood pressure (DBP), and current medication treatment (including the use of antihypertensive, hypoglycemic, and lipid-lowering drugs). Participants were considered to have hypertension if the examined SBP ≥ 140 mmHg or/and DBP ≥ 90 mmHg, offering history of hypertension, or taking antihypertensive medications (1). The definition of diabetes was detected fasting blood glucose ≥ 7.0 mmol/l (126 mg/dl), hemoglobin A1c (HbA1C) ≥6.5%, offering history of diabetes, or using hypoglycemic drugs (35).

### Outcomes

In this study, we need to observe all-cause and cardiovascular mortality as the endpoints. Participants were followed up through December 31, 2015 unless dropping out or meeting the intended outcomes. Mortality status of participants was authenticated by matches with the National Death Index. Referring to the International Classification of Diseases, 10th Edition (ICD-10), Clinical Modification System codes, cardiovascular mortality was identified as deaths caused by CVD or cerebrovascular disease (ICD-10 codes I00 to I09, I11, I13, I20 to I51, and I60 to I69).

### Statistical Analysis

Characteristics of the included participants were divided by non-HDL-C levels (<130, 130–159, 160–189, 190–219, and ≥220 mg/dl) at baseline (36), with the continuous variables marked by mean values with standard deviation (SD) and the categorical variables marked by numbers with percentages. To detect the differences between groups classified by non-HDL-C levels, one-way ANOVA, Kruskal–Wallis *H*-test, and chi-square tests were performed. Multivariate Cox regression models were applied with different adjustments. Model I was not adjusted for confounders. Model II was adjusted for age, gender, and race. Model III was adjusted for smoking, BMI, SBP, diabetes, and medication treatment in addition to the adjusted confounders of model II. Kaplan–Meier survival analyses were applied to analyze the differences of survival rates according to non-HDL-C stratification, while log-rank test was applied to examine the differences between groups. Non-linear relationships were detected by restricted cubic spline models, and then, two piecewise linear regression models were built to clarify the difference of relationships at threshold if the relationships were non-linear. The point with the highest likelihood among all the possible values was chosen to define the threshold value. The differences of the results when applying one-line or two piecewise linear regression models were compared by logarithmic likelihood ratio test. For subgroup analysis, we evaluated the results varied by age (≥65 or <65 years), gender (male vs. female), race (non-white vs. white), lipid-lowering drug usage (yes or no), and BMI (≥25 or <25 kg/m^2^) from regression analysis. We applied R version 3.6.3 (R Foundation for Statistical Computing, Vienna, Austria) to perform all the analyses, with *P* < 0.05 confirmed as statistical significance.

## Results

### Baseline Characteristics

Table 1 presents the characteristics of 12,169 participants in this study. Among all the participants, the average age was 57.27 ± 15.79 years and 47.52% were male. All baseline covariates have significant differences among non-HDL-C groups (all *P* < 0.01), except for gender, smoking status, and the outcomes. Throughout the 92.5 months of average follow-up period, 1,946 (15.99%) all-cause deaths and 422 (3.47%) cardiovascular deaths occurred.

**Table 1:** Demographic and clinical characteristics according to non-HDL-C levels.

		**Non-HDL-C, mg/dl**					
	**Total**	** <130**	**130–159**	**160–189**	**190–219**	**≥220**	***P*-value**
Number	12,169	4,082	3,498	2,551	1,318	720	
Age, years	57.27 ± 15.79	57.99 ± 16.51	57.19 ± 15.55	57.23 ± 15.37	56.61 ± 15.28	54.88 ± 14.76	<0.001
Gender—male, *n* (%)	5,783 (47.52)	1,957 (47.94)	1,607 (45.94)	1,218 (47.75)	639 (48.48)	362 (50.28)	0.163
Race—white (%)	5,412 (44.47)	1,699 (41.62)	1,619 (46.28)	1,156 (45.32)	582 (44.16)	356 (49.44)	<0.001
Smoking, *n* (%)	5,741 (47.23)	1,967 (48.22)	1,586 (45.39)	1,207 (47.41)	625 (47.49)	356 (49.44)	0.098
Diabetes, *n* (%)	3,193 (26.25)	1,308 (32.06)	797 (22.81)	574 (22.50)	322 (24.43)	192 (26.67)	<0.001
Body mass index, kg/m^2^	30.55 ± 7.17	30.22 ± 7.88	30.67 ± 7.16	30.82 ± 6.72	30.61 ± 6.28	30.81 ± 5.95	0.007
Systolic blood pressure, mmHg	136.70 ± 20.94	134.55 ± 20.78	136.21 ± 20.58	138.50 ± 21.08	139.00 ± 21.08	140.49 ± 21.37	<0.001
Diastolic blood pressure, mmHg	73.45 ± 16.02	70.69 ± 16.03	73.77 ± 15.47	74.96 ± 16.13	75.70 ± 16.08	77.75 ± 15.73	<0.001
Triglycerides							<0.001
mg/dl	150.72 ± 119.44	106.30 ± 59.79	139.96 ± 75.53	168.05 ± 94.66	208.93 ± 138.03	327.54 ± 308.08	
mmol/l	1.70 ± 1.35	1.20 ± 0.68	1.58 ± 0.85	1.90 ± 1.07	2.36 ± 1.56	3.70 ± 3.48	
Low-density lipoprotein cholesterol							<0.001
mg/dl	118.76 ± 35.90	85.06 ± 18.19	116.96 ± 14.84	141.73 ± 15.47	165.76 ± 17.17	202.08 ± 23.95	
mmol/l	3.07 ± 0.93	2.20 ± 0.47	3.02 ± 0.38	3.67 ± 0.40	4.29 ± 0.44	5.23 ± 0.62	
Total cholesterol							<0.001
mg/dl	202.60 ± 43.33	162.24 ± 24.06	197.11 ± 17.57	223.52 ± 16.62	250.92 ± 15.98	295.56 ± 40.71	
mmol/l	5.24 ± 1.12	4.20 ± 0.62	5.10 ± 0.45	5.78 ± 0.43	6.49 ± 0.41	7.64 ± 1.05	
High-density lipoprotein cholesterol							<0.001
mg/dl	52.57 ± 16.22	56.03 ± 17.71	52.87 ± 15.84	50.41 ± 14.82	48.72 ± 13.82	46.13 ± 12.94	
mmol/l	1.36 ± 0.42	1.45 ± 0.46	1.37 ± 0.41	1.30 ± 0.38	1.26 ± 0.36	1.19 ± 0.33	
Non-HDL-C							<0.001
mg/dl	150.04 ± 43.39	106.21 ± 17.53	144.24 ± 8.61	173.11 ± 8.50	202.20 ± 8.62	249.43 ± 39.17	
mmol/l	3.88 ± 1.12	2.75 ± 0.45	3.73 ± 0.22	4.48 ± 0.22	5.23 ± 0.22	6.45 ± 1.01	
Treatment, *n* (%)							
Antihypertensive drugs	6,619 (54.39)	2,480 (60.75)	1,938 (55.40)	1,301 (51.00)	602 (45.68)	298 (41.39)	<0.001
Hypoglycemic agents	1,833 (15.06)	889 (21.78)	420 (12.01)	281 (11.02)	144 (10.93)	99 (13.75)	<0.001
Lipid-lowering drugs	2,401 (19.73)	1,263 (30.94)	642 (18.35)	307 (12.03)	116 (8.80)	73 (10.14)	<0.001
Outcomes, *n* (%)							
Cardiovascular disease mortality	422 (3.47)	131 (3.21)	111 (3.17)	95 (3.72)	50 (3.79)	35 (4.86)	0.146
All-cause mortality	1,946 (15.99)	681 (16.68)	515 (14.72)	414 (16.23)	214 (16.24)	122 (16.94)	0.178

*n, number*.

*Values are mean ± standardized differences or n (%)*.

### The Relationships of Non-HDL-C With Mortality

**Figure 2:**
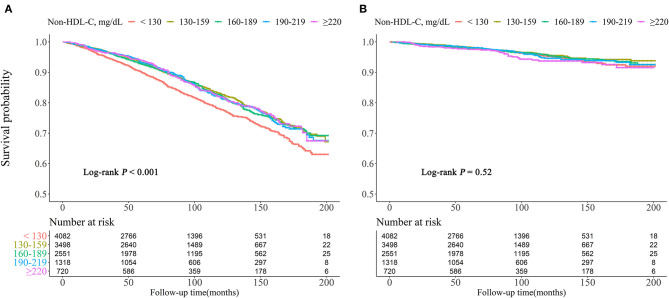
Kaplan–Meier survival curve for all-cause **(A)** and cardiovascular **(B)** mortality by non-HDL-C.

Kaplan–Meier survival curves were diverged according to non-HDL-C groups. The highest risk for all-cause mortality was observed when non-HDL-C <130 mg/dl (log rank *P* < 0.001). For cardiovascular mortality risk, there were no differences with significance among the groups (log rank *P* = 0.52) (Figure 2).

The multivariate Cox regression results are demonstrated in Table 2. When non-HDL-C was treated as a continuous variable in model III, every 10 mg/dl increment in non-HDL-C corresponded to the hazard ratio (HR) (95% confidence interval, CI) as 0.98 (0.97–0.99) for all-cause mortality and 1.02 (1.00–1.05) for cardiovascular mortality. When treating non-HDL-C as a categorical variable, compared with the reference (non-HDL-C = 130–159 mg/dl), the HR (95% CI) for all-cause death were 1.29 (1.14–1.46) when non-HDL-C <130 mg/dl and 1.01 (0.88–1.16), 1.01 (0.85–1.19), and 1.17 (0.95–1.44) for non-HDL-C levels 160–189, 190–219, and ≥220 mg/dl, respectively (*P* for trend = 0.01). Meanwhile, the HRs of cardiovascular death for non-HDL-C levels <130, 160–189, 190–219, and ≥220 mg/dl were 1.07 (0.82–1.41), 1.02 (0.76–1.36), 1.05(0.73–1.51), and 1.55 (1.03–2.34), respectively (*P* for trend = 0.284).

**Table 2:** Multivariate cox regression analysis of non-HDL-C with cause-specific mortality.

	**Event rate/ 1,000 person-** **years**	**Model I** ** HR (95% CI),** ***p*-value**	**Model II** ** HR (95% CI),** ***p*-value**	**Model III** ** HR (95% CI),** ***p*-value**
**All-cause mortality**				
Non-HDL-C (as continuous variables, per 10 mg/dl increment)	20.75	0.97 (0.96, 0.98) <0.001	0.98 (0.97, 0.99) 0.004	0.98 (0.97, 0.99) 0.003
Non-HDL-C group, mg/dl				
<130	24.19	1.33 (1.19, 1.49) <0.001	1.31 (1.17, 1.47) <0.001	1.29 (1.14, 1.46) <0.001
130–159	18.80	Reference	Reference	Reference
160–189	19.60	1.03 (0.90, 1.17) 0.672	1.04 (0.92, 1.19) 0.507	1.01 (0.88, 1.16) 0.899
190–219	19.55	1.03 (0.88, 1.20) 0.749	1.10 (0.94, 1.29) 0.249	1.01 (0.85, 1.19) 0.925
≥220	19.74	1.03 (0.84, 1.25) 0.784	1.24 (1.02, 1.51) 0.034	1.17 (0.95, 1.44) 0.135
*P* for trend		<0.001	0.036	0.01
**Cardiovascular mortality**				
Non-HDL-C (as continuous variables, per 10 mg/dl increment)	4.50	1.01 (0.99, 1.03) 0.388	1.03 (1.00, 1.05) 0.027	1.02 (1.00, 1.05) 0.1
Non-HDL-C group, mg/dl				
<130	4.65	1.16 (0.90, 1.50) 0.237	1.12 (0.87, 1.44) 0.391	1.07 (0.82, 1.41) 0.615
130–159	4.05	Reference	Reference	Reference
160–189	4.50	1.10 (0.84, 1.45) 0.479	1.13 (0.86, 1.48) 0.398	1.02 (0.76, 1.36) 0.913
190–219	4.57	1.12 (0.80, 1.57) 0.499	1.22 (0.87, 1.71) 0.239	1.05 (0.73, 1.51) 0.799
≥220	5.66	1.38 (0.95, 2.02) 0.095	1.75 (1.19, 2.55) 0.004	1.55 (1.03, 2.34) 0.037
*P* for trend		0.552	0.055	0.284

*HR, hazard ratio; CI, confidence interval*.

*Model I adjust for none; model II adjust for age, gender, and race; model III adjust for age, gender, race, smoking, body mass index, systolic blood pressure, diabetes, and medicine use (antihypertensive drugs, hypoglycemic agents, and lipid-lowering drugs)*.

### The Detection of Non-linear Relationships

**Figure 3:**
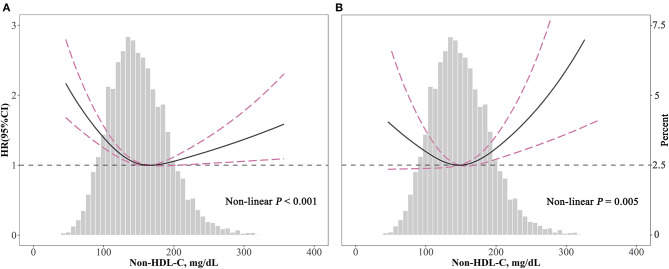
Spline analyses of all-cause **(A)** and cardiovascular **(B)** mortality by non-HDL-C in the overall cohort and the probability distribution histogram is represented in the background [spline analyses were adjusted for age, gender, race, smoking, body mass index, systolic blood pressure, diabetes, and medicine use (antihypertensive drugs, hypoglycemic agents, and lipid-lowering drugs)].

The associations of non-HDL-C with all-cause (*P* < 0.001) and cardiovascular (*P* = 0.005) mortality were non-linear and U-shaped according to restricted cubic spline models (Figure 3).

According to two piecewise linear regression models in Table 3, the cut-off values were 158 mg/dl for all-cause mortality and 190 mg/dl for cardiovascular mortality. Below the cut-off value, every 10 mg/dl increment in non-HDL-C led to lower all-cause mortality (HR = 0.94, 95% CI: 0.92–0.96), but not significantly decreased cardiovascular mortality (HR = 0.98, 95% CI: 0.95–1.02). Comparatively, above the cut-off value, for every 10 mg/dl increment, non-HDL-C promoted both all-cause (HR = 1.03, 95% CI: 1.01–1.05) and cardiovascular mortality (HR = 1.09, 95% CI: 1.05–1.14).

**Table 3:** The results of two piecewise linear regression model between non-HDL-C and cause-specific mortality.

	**All-cause mortality HR (95% CI) *P*-value**	**Cardiovascular mortality HR (95% CI) *P*-value**
Cut-off value	158	190
< Cut-off value	0.94 (0.92, 0.96) <0.001	0.98 (0.95, 1.02) 0.314
≥Cut-off value	1.03 (1.01, 1.05) 0.013	1.09 (1.05, 1.14) <0.001
*P* for log likelihood ratio test	<0.001	0.002

*HR, hazard ratio; CI, confidence interval*.

*The two piecewise linear regression models were adjusted for age, gender, race, smoking, body mass index, systolic blood pressure, diabetes, and medicine use (antihypertensive drugs, hypoglycemic agents, and lipid-lowering drugs)*.

### Subgroups Analysis

Table 4 presents the results of subgroup analysis. As for all-cause mortality, the non-linear relationships with statistical significance were found among participants who were aged <65 years old, males, non-white, not taking lipid-lowering drugs, and subjects with BMI ≥ 25 kg/m^2^. For cardiovascular mortality, the non-linear relationships with statistical significance were only found in males. When non-HDL-C ≥ 190 mg/dl, for every 10 mg/dl increment, non-HDL-C elevated cardiovascular mortality risk significantly in participants who were aged <65 years old (HR = 1.11, 95% CI: 1.07–1.15), males (HR = 1.12, 95% CI: 1.07–1.17), non-white (HR = 1.11, 95% CI: 1.06–1.17), not taking lipid-lowering drugs (HR = 1.09, 95% CI: 1.04–1.14) or taking lipid-lowering drugs (HR = 1.11, 95% CI: 1.01–1.22), and individuals with BMI ≥ 25 kg/m^2^ (HR = 1.10, 95% CI: 1.05–1.15).

**Table 4:** Subgroup analysis.

	***N***	**All-cause mortality HR (95% CI)** ***P*****-value**		***P* for log likelihood ratio test**	**Cardiovascular mortality HR (95% CI)** ***P*****-value**		***P* for log likelihood ratio test**
**Cutoff value, mmol/l**		** <158**	**≥158**		** <190**	**≥190**	
**Age**							
≥65	4,323	0.95 (0.93, 0.98) <0.001	1.00 (0.98, 1.03) 0.769	0.021	1.00 (0.96, 1.04) 0.896	1.02 (0.94, 1.11) 0.669	0.777
<65	7,846	0.93 (0.89, 0.97) <0.001	1.03 (1.01, 1.06) 0.018	<0.001	0.95 (0.89, 1.02) 0.164	1.11 (1.07, 1.15) <0.001	0.002
**Gender**							
Male	5,783	0.91 (0.88, 0.93) <0.001	1.04 (1.01, 1.07) 0.014	<0.001	0.95 (0.91, 1.00) 0.046	1.12 (1.07, 1.17) <0.001	<0.001
Female	6,386	0.97 (0.94, 1.01) 0.117	1.01 (0.98, 1.04) 0.396	0.149	1.01 (0.96, 1.07) 0.63	1.03 (0.94, 1.13) 0.517	0.787
**Race**							
Non-white	6,757	0.93 (0.90, 0.96) <0.001	1.04 (1.01, 1.07) 0.003	<0.001	0.97 (0.93, 1.02) 0.204	1.11 (1.06, 1.17) <0.001	0.002
White	5,412	0.95 (0.92, 0.98) <0.001	1.01 (0.98, 1.04) 0.462	0.015	1.00 (0.95, 1.05) 0.999	1.06 (0.98, 1.15) 0.157	0.327
**Lipid-lowering drugs**							
No	9,768	0.93 (0.91, 0.96) <0.001	1.02 (1.00, 1.05) 0.034	<0.001	0.98 (0.95, 1.02) 0.397	1.09 (1.04, 1.14) <0.001	0.013
Yes	2,401	0.96 (0.91, 1.00) 0.061	1.03 (0.98, 1.09) 0.25	0.086	0.99 (0.91, 1.07) 0.779	1.11 (1.01, 1.22) 0.024	0.136
**Body mass index, kg/m**^**2**^							
<25	2,422	0.92 (0.88, 0.95) <0.001	1.03 (0.98, 1.08) 0.227	0.002	0.98 (0.93, 1.05) 0.6	1.07 (0.93, 1.23) 0.323	0.346
≥25	9,491	0.95 (0.92, 0.98) <0.001	1.03 (1.00, 1.05) 0.023	<0.001	0.99 (0.95, 1.03) 0.699	1.10 (1.05, 1.15) <0.001	0.009

*When analyzing a subgroup variable, age, gender, race, smoking, body mass index, systolic blood pressure, diabetes, and medicine use (antihypertensive drugs, hypoglycemic agents, and lipid-lowering drugs) were all adjusted except the variable itself*.

## Discussion

The results in this study presented that non-HDL-C had a U-shaped association with all-cause and cardiovascular mortality in hypertensive population. The lowest risk was found at threshold values of 158 and 190 mg/dl for all-cause and cardiovascular mortality, respectively. Relatively higher or lower non-HDL-C concentrations linked to increased mortality.

One of our major findings is that higher non-HDL-C led to higher mortality, which is similar to previous research findings in general population and CVD patients (14–19). Some possible explanations for these results were listed below. First, non-HDL-C could represent all the atherogenic lipoproteins. These lipoproteins could accumulate in the arterial intima, causing the formation of atherosclerotic plaques (37). Atherosclerosis caused by high non-HDL-C can increase morbidity and mortality of atherosclerotic cardiovascular disease (ASCVD). Therefore, non-HDL-C is widely advocated for cardiovascular risk stratification (13). Second, the mutations of some genes like LDLR, apoB, and PCSK9 can lead to hypercholesterolemia. Patients with hypercholesterolemia have increased non-HDL-C and are more prone to suffer from ASCVD and cardiovascular death (38). The 2013 ACC/AHA guidelines suggested that non-HDL-C ≥ 220 mg/dl could possibly imply hereditary genetic hypercholesterolemia, requiring additional clinical examinations (39). Third, our study was conducted in patients with hypertension, who have relatively higher non-HDL-C levels than the healthy population (31). Previous studies revealed that hypertension is related to dyslipidemia and may promote each other. Bonaa et al. evaluated the cross-sectional relationships between lipid levels and blood pressure and found a positive association (2). In the prospective study of Otsuka et al., 14,215 men without hypertension (aged 38 ± 9 years) were enrolled, and 1,483 subjects developed hypertension during 4 years. After adjusting for confounding factors, they also found that subjects having higher non-HDL-C, LDL-C, and TC suffered greater hypertension risk, while HDL-C was U-shaped associated with hypertension risk (40). The cross-sectional study of Lautsch et al. revealed the link of elevated non-HDL-C with raised blood pressure in hypertensive patients (30). The meta-analysis by Strazzullo et al. explored how statin treatment affects blood pressure, and the results showed that the blood pressure of patients taking statins was lower than those taking placebo and statins had a greater effect on higher baseline blood pressure (41). It indicated that lipid-lowering therapy may promote the control of blood pressure. From another perspective, there is a positive correlation between non-HDL-C and arterial stiffness in hypertensive patients according to the studies of Zhan et al. and Wen et al. (28, 29). Several mechanisms could explicate. For example, hypercholesterolemia can stimulate the renin–angiotensin–aldosterone system, increasing levels and activity of Ang I, Ang II, and endothelin-1, which may lead to the elevation of blood pressure (42). Besides, hypercholesterolemia and hypertension can affect endothelial cells through a common signaling pathway, leading to endothelial dysfunction (43). In brief, elevated non-HDL-C could be a factor causing poor blood pressure control and damaged vascular function in hypertensive population, resulting in poor prognosis and higher mortality.

We also observed that lower non-HDL-C linked to higher mortality risk in hypertensive population. Consistent with our results, Chiu et al. also found the U-shaped association in chronic kidney disease patients; the lowest mortality risk was observed at non-HDL-C levels of 116.2–143.9 mg/dl, and non-HDL-C <116.2 mg/dl appeared with higher mortality (27). Non-HDL-C was inversely associated with mortality among hemodialysis patients in the analysis of Chang et al. (24). They classified the study population by non-HDL-C levels (<60, 60–85, 85–100, 100–115, 115–130, 130–145, 145–160, and ≥160 mg/dl), which were relatively lower than other relevant studies. Bathum et al. and Tuikkala et al. found an inverse association of cholesterol with mortality in their studies of the elderly (25, 26). The studies above have mentioned a poorer health status in patients with lower cholesterol levels. Though we have excluded patients with cancer, there could be other non-cardiovascular comorbidities or variables that indicated poor health that we have not adjusted for. Thus, low non-HDL-C levels still could represent the frailty in our study population; malnutrition and inflammation that lead to further aggravation in debilitated patients may explain such inverse association (44). In addition, below the threshold value, we found the increase of all-cause mortality more significant than that of cardiovascular mortality with the decrease of non-HDL-C, which could be ascribed to higher non-cardiovascular deaths in those feeble patients (45). Comparatively, higher non-HDL-C resulted in higher cardiovascular mortality, but lower non-cardiovascular mortality (46), which might explain the greater propensity of developing cardiovascular deaths than all-cause deaths above the threshold value we observed. Furthermore, according to the calculation formula, relatively low non-HDL-C levels could represent lower TC or higher HDL-C. Our previous studies found extremely high HDL-C increasing mortality in general or hypertensive population by analyzing the data from NHANES (47, 48). Other relevant studies also showed similar results (49, 50). This finding might be resulted from the genetic variation of particular genes (51) and variation of the size or function of HDL particles (52).

Paradoxically, some studies found out that non-HDL-C was not associated with mortality (20, 21). The reason for such contradiction may be due to different adjusted confounding factors or various enrolled populations in these studies. In the present study, the subgroup analysis showed the non-linear relationships with statistical significance only among certain subgroups. It was indicated that different classifications of subgroups would affect the results, especially the stratification according to age and gender. Non-HDL-C levels in the subgroup <65 years old showed a stronger correlation with mortality than the subgroup ≥65 years old, and the non-linear relationship is more significant in men than in women. In line with our findings, a large multinational risk-evaluation study found subjects under 45 years old have higher long-term risk of CVD in association with non-HDL-C, and men suffered from about 13% greater probability of experiencing CVD events than women in the same range of non-HDL-C levels (10). Such divergences may be given to the shorter lifespan in elderly (10), estrogen-induced cholesterol reduction, and prevention of blood vessels in premenopausal women (53). The elderly may reach the mortality endpoints due to the age itself, while the women were less likely to suffer from the cholesterol-related deaths. Our subgroup analysis indicated that non-HDL-C may predict mortality better in men and young or middle-aged people, which suggested that lipid-lowering is of especial value in preventing cardiovascular risk and mortality in hypertensive patients of these categories.

### Study Strength and Limitations

Our study was linked to a national representative database with reasonable design and techniques, which may provide reference for blood lipid assessment and mortality risk evaluation in hypertensive population. However, some limitations of this study should be considered. First, some variables included in this study like smoking status and current medication treatment were subjective from participants, which may cause recall bias. Second, the blood lipid levels were only measured once, which may be inaccurate, because the lipid levels may change during the follow-up time. An analysis using NHANES data showed that for 4,226 individuals with complete blood lipid data, non-HDL-C fell by 21% after 17 years (54). Third, findings in this study are applicable to the population in North America and probably not applicable to the population in other regions.

## Conclusion

In this study, non-HDL-C was U-shaped associated with mortality among patients with hypertension. Further studies are needed to confirm our findings so as to verify the values of using non-HDL-C for risk stratification in hypertensive population.

## Data Availability Statement

Publicly available datasets were analyzed in this study. This data can be found here: https://www.cdc.gov/nchs/nhanes/index.htm.

## Ethics Statement

The studies involving human participants were reviewed and approved by The Centers for Disease Control and Prevention. The patients/participants provided their written informed consent to participate in this study. Written informed consent was obtained from the individual(s) for the publication of any potentially identifiable images or data included in this article.

## Author Contributions

Y-qH, X-cL, and QC formed the study concept and interpreted the data. X-cL analyzed the data. C-lC, Y-qF, and J-yC supervised this study. QC composed and revised the manuscript. All authors made contributions to this work and agreed the final edition.

## Conflict of Interest

The authors declare that the research was conducted in the absence of any commercial or financial relationships that could be construed as a potential conflict of interest.
